# *Trichoderma* – genomes and genomics as treasure troves for research towards biology, biotechnology and agriculture

**DOI:** 10.3389/ffunb.2022.1002161

**Published:** 2022-09-14

**Authors:** Miriam Schalamun, Monika Schmoll

**Affiliations:** ^1^ Center for Health and Bioresources, AIT Austrian Institute of Technology GmbH, Tulln, Austria; ^2^ Department of Microbiology and Ecosystem Science, Division of Terrestrial Ecosystem Research, University of Vienna, Vienna, Austria

**Keywords:** *Trichoderma*, *Hypocrea*, evolution, horizontal gene transfer, repeat induced point mutation, mycovirus, bioremediation, biocontrol

## Abstract

The genus *Trichoderma* is among the best studied groups of filamentous fungi, largely because of its high relevance in applications from agriculture to enzyme biosynthesis to biofuel production. However, the physiological competences of these fungi, that led to these beneficial applications are intriguing also from a scientific and ecological point of view. This review therefore summarizes recent developments in studies of fungal genomes, updates on previously started genome annotation efforts and novel discoveries as well as efforts towards bioprospecting for enzymes and bioactive compounds such as cellulases, enzymes degrading xenobiotics and metabolites with potential pharmaceutical value. Thereby insights are provided into genomes, mitochondrial genomes and genomes of mycoviruses of *Trichoderma* strains relevant for enzyme production, biocontrol and mycoremediation. In several cases, production of bioactive compounds could be associated with responsible genes or clusters and bioremediation capabilities could be supported or predicted using genome information. Insights into evolution of the genus *Trichoderma* revealed large scale horizontal gene transfer, predominantly of CAZyme genes, but also secondary metabolite clusters. Investigation of sexual development showed that *Trichoderma* species are competent of repeat induced point mutation (RIP) and in some cases, segmental aneuploidy was observed. Some random mutants finally gave away their crucial mutations like *T. reesei* QM9978 and QM9136 and the fertility defect of QM6a was traced back to its gene defect. The *Trichoderma* core genome was narrowed down to 7000 genes and gene clustering was investigated in the genomes of multiple species. Finally, recent developments in application of CRISPR/Cas9 in *Trichoderma*, cloning and expression strategies for the workhorse *T. reesei* as well as the use genome mining tools for bioprospecting *Trichoderma* are highlighted. The intriguing new findings on evolution, genomics and physiology highlight emerging trends and illustrate worthwhile perspectives in diverse fields of research with *Trichoderma*.

## Introduction

The genus *Trichoderma* belongs to the most beneficial group of fungi for humanity, which explains the extensive research efforts dedicated to biology and biotechnology with these fungi (Schuster and Schmoll, 2010; Druzhinina et al., 2011; Bischof et al., 2016; Guzman-Guzman et al., 2019). While industrial application of *Trichoderma* for protein production is limited to the descendants of a single species (Bischof et al., 2016; Paloheimo et al., 2016; Arnau et al., 2020), applications in agriculture for biocontrol and plant protection involve numerous species and strains (Kashyap et al., 2017; Lahlali et al., 2022; Tyskiewicz et al., 2022) and their high performance made *Trichoderma* the biocontrol agent with the highest market performance in terms of value, even higher than bacterial biocontrol agents together^1^ ^2^. Due to their versatility, *Trichoderma* species serve as models for such important topics like mechanisms regulating plant cell wall degradation (Glass et al., 2013; Bischof et al., 2016), biocontrol (Harman et al., 2004a; Guzman-Guzman et al., 2019; Harman et al., 2021), effector like molecules (Ramirez-Valdespino et al., 2019) and light response (Schmoll et al., 2010; Carreras-Villaseñor et al., 2012; Schmoll, 2018a; 2018b). Additionally, *Trichoderma* spp. are a valuable source for natural products leveraged by screening genomes with constantly enhanced software tools (Rush et al., 2021). Recently, even a connection between the innate immune system of animals and *Trichoderma* was drawn (Medina-Castellanos et al., 2018). Interestingly, *Trichoderma* can initiate heritable plant priming responses, which are attributed to epigenetic regulation (Moran-Diez et al., 2021). Not only the active fungi themselves, but also extracts of *Trichoderma* spp. can inhibit growth and/or production of mycotoxins by pathogens (Stracquadanio et al., 2021). However, only a few years ago also a potential downside of the distribution of *Trichoderma* in nature became obvious with the detection of *T. afroharzianum* causing maize ear rot disease (Pfordt et al., 2020; Sanna et al., 2022). Consequently, species identification and genomic competences of these fungi deserve particular attention. *Trichoderma* spp. show an exceptional versatility in their preferred habitats and substrates with lifestyles ranging from mycoparasitism to plant saprotrophy and accordingly life in habitats characterized by feeding on fungi or decaying plant material, in soil or even as endophytes in living plant tissue, which changed during evolution several times (Chaverri and Samuels, 2013)

**Figure 1:**
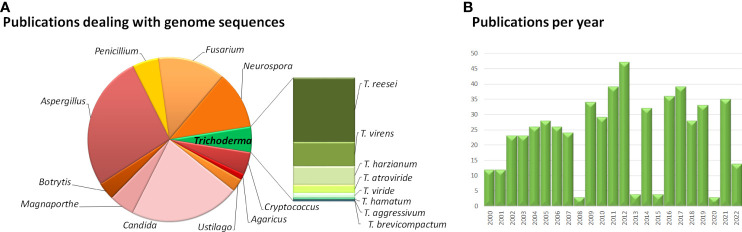
Overview of studies dealing with fungal genomes. **(A)** Results of a Pubmed search for "genome sequences” of the respective fungi. **(B)** Number of studies dealing with „*Trichoderma* genome sequences” per year published in Pubmed. The website pubmed.ncbi.nlm.nih.gov/was accessed in August 2022.

Starting from the genome sequence of *T. reesei*, which was published in 2008 (Martinez et al., 2008), the genomes of numerous *Trichoderma* model strains for enzyme production and biocontrol followed (**Figure 1**). The availability of the genome sequences of major model fungi of *Trichoderma* considerably contributed to detailed investigation of mechanisms of action and regulation of pathways (Sood et al., 2020), which is focused on systemic resistance of plants (Shoresh et al., 2010), colonization (Hinterdobler et al., 2021b; Taylor et al., 2021; Hafiz et al., 2022; Taylor et al., 2022), effector molecules (Ramirez-Valdespino et al., 2019) and plant-fungus-pathogen interactions (Mendoza-Mendoza et al., 2018; Macias-Rodriguez et al., 2020; Alfiky and Weisskopf, 2021) among others.

One crucial task in the future will be to seriously investigate both competences of *Trichoderma* strains that can be applied for human benefit and to balance these benefits with potential threats by strains with harmful characteristics for human and plant health. The imminent climate crisis and the aim of more sustainable and safe agriculture require increased research efforts to safely develop biocontrol applications and support of plant health by microbes without risking ecological or human adversities.

The NCBI taxonomy browser (https://www.ncbi.nlm.nih.gov/Taxonomy/Browser/; accessed on July 11, 2022) lists more than 400 *Trichoderma* species and additionally over 1800 unclassified *Trichoderma* species. The ecophysiology and evolution of a first batch of more than 30 *Trichoderma* species is currently subject to a large scale sequencing effort with the Joint Genome Institute (JGI community sequencing project CSP-503464), which will be followed by analyses of another several hundred species to be analyzed.

Large scale research efforts for many species of *Trichoderma* revealed the evolutionary basis of the versatility of the fungi in this genus and provided important insights into their evolution. The genus evolved about 66 million years ago with later formation of the sections Longibrachiatum and Trichoderma and the clade Harzianum/Virens (Kubicek et al., 2019). Evolution of the genus was dominated by considerable gene gain (predominantly encoding ankyrins, HET domain proteins and transcription factors) and loss events around a core genome of exactly 7000 genes, which formed the basis for the diverse competences of *Trichoderma* (Kubicek et al., 2019). This core genome is dominated by genes involved in metabolism, with an important share of genes assigned to the KOG families “posttranslational modification, protein turnover and chaperones”, “transcription” and “carbohydrate transport and metabolism”, but also high numbers of glycoside hydrolases and fungal specific transcription factors (Kubicek et al., 2019). The constant adaptation and optimization of *Trichoderma* genomes is reflected in the finding that of the 105 orphan genes found in the core *Trichoderma* genome by comparing fungi out of this genus, most are under strong purifying selection (Kubicek et al., 2019).

## *Trichoderma* – fungal pirates with a taste for plants

Being among the most important genera for industry and agriculture, the evolution of *Trichoderma* and their metabolic consequences is highly relevant for research. In recent years, enabled by sequencing efforts for several important *Trichoderma* species, intriguing new aspects on evolution in the genus *Trichoderma* were revealed, which in part explain their special characteristics. Horizontal or lateral gene transfer (HGT/LGT) has long been known to play a role in evolution, also in fungi, although it was considered to be more important in bacteria at first (Wang et al., 2015b). However, discrepancies of gene content or presence of whole clusters in closely related species can also be due to selective pressure and loss of genes, or a combination of selection and HGT (Hou et al., 2022).

**Figure 2:**
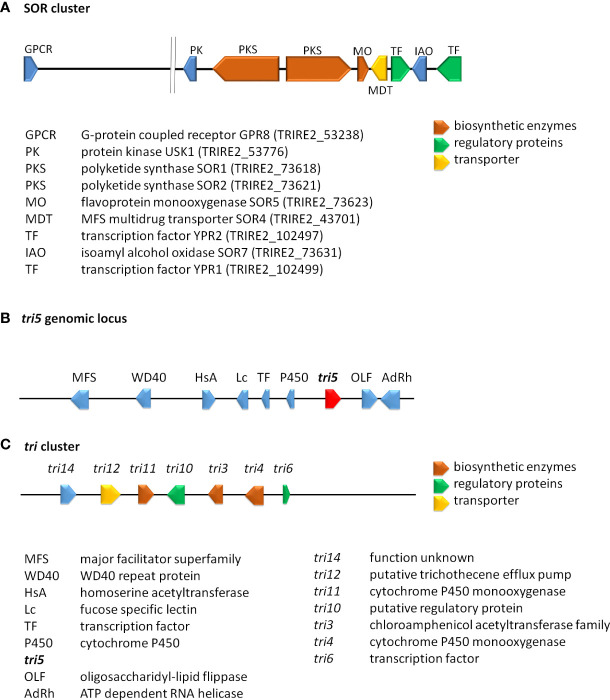
Schematic representation of selected secondary metabolite clusters in *Trichoderma*. **(A)** The SOR cluster in *T. reesei* along with characterized flanking genes. Functions and JGI protein IDs (*T. reesei* v2.0) are listed below the scheme. **(B)** The genomic locus of *tri5* in *T. arundinaceum* and *T. brevicompactum* (Gutierrez et al., 2021). **(C)** The *tri* cluster in *T. arundinaceum* (Cardoza et al., 2011). Encoded proteins of the *tri* cluster and the *tri5* genomic locus are given below the scheme.

An intriguing example for the latter phenomenon was found with the sorbicillin secondary metabolite gene cluster in *T. reesei* (**Figure 2**) (Druzhinina et al., 2016). Sorbicillinoids are yellow to orange secondary metabolites (Derntl et al., 2016; Salo et al., 2016; Guzman-Chavez et al., 2017), with weak antagonistic effects against bacteria (Duan et al., 2022; Hou et al., 2022) and pharmacological activity (Meng et al., 2016). The respective gene cluster contains two polyketide synthases, an FAD dependent monooxygenase, an MSF transporter and two transcription factors as most important components (Druzhinina et al., 2016; Monroy et al., 2017). The cluster is transcriptionally regulated by light and in response to different carbon sources (Monroy et al., 2017; Stappler et al., 2017) as well as by the two transcription factors in the cluster, YPR1 (Derntl et al., 2016; Derntl et al., 2017) and YPR2 (Hitzenhammer et al., 2019), by the carbon catabolite repressor CRE1 (Monroy et al., 2017) and by LAE1 (Karimi Aghcheh et al., 2013).

The genes of this cluster are only in part present in other *Trichoderma* spp, but closely related to clusters in *Penicillium notatum* and other Eurotiomycetes as well as a few Sordariomycetes (Maskey et al., 2005; Harned and Volp, 2011) and their phylogeny is not in accordance with the Ascomycota phylogeny, which hints at HGT events from different ancestors. Investigation of the evolution of the SOR cluster revealed several HGT events between *Acremonium chrysogenum, Penicillium rubens, Ustilaginoidea virens, Colletotrichum graminicola* and *Trichoderma*, in which *Trichoderma* spp. appears to only have received SOR2 (Druzhinina et al., 2016). To explain the evolution of the remaining genes of the cluster, selection and gene loss were analyzed, which showed that the cluster arose in early Hypocreales, was complemented by HGT and is under strong purifying selection (Druzhinina et al., 2016). In *T. reesei* two additional genes with clear relations to the cluster and located close to it in the genome were detected. The gene encoding protein kinase USK1 (unique SOR cluster kinase) is located only 2.3 kb upstream of *sor1* and is not syntenic in other fungi, not even in *Trichoderma* spp. (Beier et al., 2020). USK1 regulates VEL1 which is crucial for coordination of secondary metabolism with development (Bayram and Braus, 2012; Bazafkan et al., 2015) and several genes of the SOR cluster. Accordingly, lack of USK1 decreases production of sorbicillins considerably, but is also required for normal levels of alamethicine and paracelsins (Beier et al., 2020).

In a distance of about 60 kb upstream of the SOR cluster, the G-protein coupled receptor GPR8 (Hinterdobler et al., 2020a) is located, which is largely co-regulated with the SOR cluster under several conditions (Stappler et al., 2017). Although homologues of GPR8 are encoded in other *Trichoderma* spp. and related fungi, their locus in the genome is not syntenic and the homologous genes are mostly located on different scaffolds or chromosomes (Hinterdobler et al., 2020a). GPR8 is the only member of class VII (secretin like) G-protein coupled receptors in the currently investigated *Trichoderma* spp. (Gruber et al., 2013; Schmoll et al., 2016). While the ligand of GPR8 is not yet known, transcriptome analysis showed a considerable impact on gene regulation predominantly in darkness, which not only impacts secondary metabolism, but also carbon metabolism (Hinterdobler et al., 2020a). In accordance with its impact on the SOR cluster genes, the regulatory targets of GPR8 overlap also with those of the SOR cluster transcription factor YPR2 (Hitzenhammer et al., 2019) and production of sorbicillinoids, alamethicine, orsellinic acid and paracelsins is strongly decreased (Hinterdobler et al., 2020a). Despite their clear relations to the SOR cluster, the phylogenetic characteristics of both GPR8 and USK1 do not align with the phylogeny of *Trichoderma* within the ascomycetes, which hints at an involvement of either HGT or selection pressure on these genes during evolution as shown for the SOR cluster genes, which remains to be investigated.

In summary, the question remains, why this cluster was retained and even complemented during evolution in *T. reesei*, but not in closely related species. Since sorbicillins only have a relatively weak antagonistic function towards other microbes (Derntl et al., 2017), it is unlikely that their major relevance lies in competition and defense. Interestingly, transcriptome analyses revealed that the core SOR cluster genes are among those most strongly transcribed under conditions of sexual development in *T. reesei* (Dattenböck et al., 2018). Additionally, mutual transcriptional regulation of *sor1*, *sor2* and *sor5* establishes a pattern reminiscent of a feedback cycle, which acts positively in light and negatively in darkness (Monroy et al., 2017). Since deletion of *gpr8* resulted in altered regulation of several sensing and signaling genes including eight G-protein coupled receptor genes, the function of sorbicillinoids as signaling molecules and of the SOR cluster as a tool for communication warrants further investigations.

A history of gene loss and potential re-acquisition *via* HGT was also proposed for a gene involved in trichothecene production. Trichothecenes are harmful mycotoxins, which are produced by several genera of the fungal order Hypocreales (Cardoza et al., 2011). The terpene synthase gene *tri5* (Malmierca et al., 2013), which catalyzes formation of trichodiene is present in a part of *Trichoderma* species, but not in all cases with a functional copy (Vicente et al., 2020) (Gutierrez et al., 2021). In contrast to other fungi, the gene in *Trichoderma* is located outside of the trichothecene biosynthesis cluster (**Figure 2**) (Lindo et al., 2018; Proctor et al., 2018). This interesting evolution of gain and loss of *tri5*, sometimes even if the associated trichothecene biosynthesis cluster was not present in the genome, suggests a competitive advantage of trichodiene production, justifying the acquisition of the whole and parts of the cluster (Gutierrez et al., 2021).

A further case for the occurrence of LGT is the group of ceratoplatanins with three members in the core genome of *Trichoderma* (some *Trichoderma* species have also 4 ceratoplatanin genes) of which only one is efficiently expressed (Gaderer et al., 2014; Gaderer et al., 2015; Gao et al., 2020). Ceratoplatanins of *Trichoderma* are involved in the activation of plant defences and induce expression of peroxidase and dioxygenase encoding genes (Salas-Marina et al., 2015), reduce surface hydrophobicity and negatively affect root colonization (Gao et al., 2020). Analysis of evolution of EPL1-like ceratoplatanins in 37 *Trichoderma* genomes and numerous other genomes revealed their likely origin in Basidiomycota with a distribution to Ascomycota by several ancient events of LGT (also within Ascomycota) and subsequent diversification by birth-and-death evolution. Moreover, all four *Trichoderma* ceratoplatanins were found to be under purifying selection pressure (Gao et al., 2020).

For the evolution of hydrophobins, which form hydrophobic layers that cover fungal bodies and spores (Bayry et al., 2012; Guzman-Guzman et al., 2017) and broadly affect fungal physiology (Cai et al., 2021) a differential impact on fungal fitness was proposed (Cai et al., 2020). Especially *T. harzianum* HFB4 was shown to evolve under strong positive selective pressure, while most other hydrophobins were subject to purifying selection. Consequently, evolution of hydrophobins is suggested as an example of fitness tradeoffs during evolution (Kubicek et al., 2008; Cai et al., 2020).

However, the genomes of *Trichoderma* hold remnants of even more intriguing events, which shaped their physiological characteristics. *Trichoderma* species are known for their exceptional abilities to feed on a broad range of substrates from plant debris to dead fungi to parasitism on living pathogens and diverse degradation products related to these materials (Druzhinina et al., 2011; Druzhinina et al., 2018). Although at first different species were considered specialists for either degradation of cellulosic plant material or fungal biomass (Kubicek 2011), later studies showed that these metabolic competences are present in all *Trichoderma* species (Druzhinina and Kubicek, 2013). In fact, *Trichoderma* shares a last common ancestor with entomoparasitic fungi and the phylogenetic branch leading to the plant associated Nectriaceae diverged earlier in evolution (Druzhinina et al., 2018). Intriguingly, in depth analysis of nine *Trichoderma* genomes revealed that more than 40% of the plant cell wall degrading genes encoding CAZymes (Carbohydrate Active enZymes) of *Trichoderma* was gathered from other plant associated filamentous Ascomycota by LGT. The auxiliary protein swollenin (Saloheimo et al., 2002; Brotman et al., 2008) was even likely obtained from green plants by LGT (Druzhinina et al., 2018), which is supported by the finding that *Trichoderma* spp. are frequently found as members of endophytic fungal communities (Gazis and Chaverri, 2010; Yadav et al., 2022) and generally plant symbionts living in their rhizosphere (Harman et al., 2004a). In contrast to numerous genes encoding enzymes, their major regulators XYR1, ACE2 and ACE3 (Benocci et al., 2017) evolved by vertical gene transfer, but not by LGT (Druzhinina et al., 2018). The results of this study further suggest that *Trichoderma* spp. originally represented potent mycoparasites and that this very lifestyle allowed them to broaden their metabolic competences by collecting, combining and optimizing novel genes they encountered when feeding on fungal prey. Hence the plant cell wall degradation related CAZome of *Trichoderma* is distinct from other hypocrealean fungi. In addition to genes extending the nutrient versatility of *Trichoderma*, 123 further genes were detected, which were putatively obtained by other fungi. They include genes encoding four GPCRs of the PTH11 type, two of which were tested for a relevance in cellulase regulation previously (Stappler et al., 2017), the small unique protein OOC1 (Schmoll and Kubicek, 2005; Pang et al., 2021), the dehydrogenase GRD1, which impacts cellulase regulation in a light dependent manner (Schuster et al., 2011), the high affinity nitrate transporter NIT10, CIP1, which was identified as one of the VIP genes limiting hydrolysis performance of cellulase mixtures (Lehmann et al., 2016) and CON-13, which is putatively involved in asexual reproduction (Hager and Yanofsky, 1990).

With respect to CAZymes and polysaccharide degradation, the description of a novel glucuronan lyase system in *T. parareesei* (Druzhinina et al., 2010) adds a further highlight to the versatility of the genus (Pilgaard et al., 2022). Detailed analyses of activity and degradation products confirmed that this degradation system enables complete degradation of glucoronan, which is found in fungi like *Agaricus bisporus* – a possible prey of the mycoparasite *T. parareesei*, to easily absorbable dimers and monomers (Pilgaard et al., 2022).

A further interesting aspect of evolution was reported for the processivity of cellulases with *T. reesei* CEL6A and CEL7a as examples. While in fungi, family 7 glycoside hydrolases are highly processive, the same characteristic is adopted by bacteria for family 6 cellulases. In both cases, the presence of highly processive and less processive cellulases avoids “traffic jams” on the cellulose fibers, which would decrease efficiency. Consequently, the high relevance of efficient cellulose degradation for competitive success in nature obviously led to convergent evolution of cellulases in which the structural features of cellulases was shaped to optimize processivity and interplay of the cellulose degrading machinery in bacteria and fungi (Uchiyama et al., 2020).

## Recombination and mating as tools of evolution - repeat induced point mutation and segmental aneuploidy in *Trichoderma*

Sexual development is crucial for propagation and evolution of a species (Bennett and Turgeon, 2016; Wallen and Perlin, 2018), especially if environmental conditions deteriorate (Debuchy et al., 2010; McDonald et al., 2016). In *Trichoderma*, numerous strains of diverse species were isolated from fruiting bodies (Jaklitsch and Voglmayr, 2015) – indicating that mating happens in nature – but only for *T. reesei* induction of sexual development was achieved under laboratory conditions (Seidl et al., 2009; Hinterdobler et al., 2020b). Although this novel tool for strain improvement opens up intriguing perspectives for industry, in that strains can be bred for enhanced performance, also drawbacks became obvious. Besides the female sterility of QM6a, which is due to a defect in the scaffolding protein HAM5 (Seidl et al., 2009; Linke et al., 2015; Tisch et al., 2017), the process of repeat induced point mutation may hamper optimization of genetically already modified production strains.

RIP was first discovered in *Neurospora* (Selker et al., 1987) as a mechanism for protection of the genome from transposable elements and mobile DNA (Gladyshev, 2017) and is operative in most Ascomycota (van Wyk et al., 2020). In case of a functional RIP mechanism, repetitive DNA sequences >400 bp cause C-5 cytosine methylation and deamination by the methyltransferases *rid1* and *dim2* prior to meiosis (Kouzminova and Selker, 2001; Li et al., 2018). Nevertheless, RIP was also observed for smaller duplicated regions (Gladyshev and Kleckner, 2014).

Genomes of organisms in which RIP is operative show a low number of repetitive sequences or transposon remnants (Gladyshev, 2017) as for example *N. crassa* (Krumlauf and Marzluf, 1980; Borkovich et al., 2004), which is also true for the genome of *T. reesei* (Martinez et al., 2008) although RIP was not unequivocally detected at first (Schuster et al., 2012). Nevertheless, operation of RIP is different in *T. reesei* compared to *N. crassa* (Li et al., 2018) with different dinucleotide preference and requirement of the methyltransferase genes *rid1* and *dim2* for normal sexual development in *T. reesei* but not *N. crassa*. Interestingly, *T. reesei* QM6a was found to have comparatively few transposon sequences, which are mostly located in AT-rich regions (Li et al., 2017).

Consequently, this mechanism is of utmost importance for evolution, especially considering the history of lateral gene transfer as well as gain and loss of genes as seen in *Trichoderma*. Additionally, evolution of the genome by gene duplication would be counteracted in a sexually propagating population of *Trichoderma*.

Besides RIP, another phenomenon decreased the enthusiasm after the achievement of sexual development under laboratory conditions. Already in the first crosses of QM6a with the fertile CBS999.97 (MAT1-1) strain, the diverse phenotypes of the progeny were obvious (M. Schmoll, unpublished). Detailed analysis then showed that of the 16 ascospores generated by meiosis and two rounds of postmeiotic mitosis four to eight were inviable and segmentally aneuploid ascospores were found (Chuang et al., 2015; Schmoll and Wang, 2016). The segmental duplication and deletion in the respective genome area caused loss of the polyketide synthase *pks4*, which is responsible for spore coloration (Atanasova et al., 2013) and loss of a xylanase and hence lignocellulosic biomass degradation efficiency increased in these progeny (Chuang et al., 2015). However, this process is not easily predictable in crosses between other nature isolates of *T. reesei* or among production strains.

## High quality genome sequences of important plant symbionts

Besides strains of *T. reesei*, also strains of prototypical biocontrol agents were recently re-sequenced to get high quality genome sequences for reliable analysis of genome synteny and evolutionary events (Li et al., 2021b). This analysis revealed that the telomeric sequences are conserved between *T. reesei* CBS999.97, *T. reesei* QM6a, *T. virens* Gv29-8, *T. virens* FT-333, *T. atroviride* P1 and *T. asperellum* FT-101. Third generation sequencing now also enabled analysis of AT-rich blocks, which can hardly be aligned based on the short NGS-sequence reads. Interestingly, the *T. reesei* strains have a lower percentage of AT-rich blocks than the four other strains (7-8% versus 11-13%), which in part explains their larger genome sizes (Li et al., 2021b). Generally, the genome contents of the four *Trichoderma* species are highly divergent, with 2000 – 3000 species specific genes each and obviously all seven tested high quality *Trichoderma* genomes underwent extensive transposon invasions followed by RIP mutations. Additionally, evidence for considerable chromosome shuffling in *Trichoderma* was found (Li et al., 2021b). In addition to the previously reported 7000 core *Trichoderma* genes (Druzhinina et al., 2018), the four biocontrol agents possess 800 more conserved genes (Li et al., 2021b). An important divergence in gene content was observed for transcription factor genes, with variations in the subfamilies of fungal zinc-binuclear cluster domains and fungal specific transcription factor domains, while the gene numbers of the other transcription factor families were identical among the seven genomes. Moreover, the investigated high quality genome sequences support the hypothesis that CAZyme genes form physically linked CAZyme gene clusters in polysaccharide utilization loci. Besides several species specific clusters, *T. reesei* and *T. virens* were found to share 14 common CAZyme gene clusters whereas *T. atroviride* and *T. asperellum* share 18 (Li et al., 2021b). As for secondary metabolite gene clusters, *T. reesei* and *T. virens* share 27 common clusters and *T. atroviride* and *T*. *asperellum* have 37 clusters in common (Li et al., 2021b).

The frequent presence of genes associated with secondary metabolism in such clusters was already noted with the first genome analysis of *T. reesei* and also specific secondary metabolite clusters were detected (Martinez et al., 2008). Investigation of the distribution and evolution of Cytochrome P450 genes in several *Trichoderma* spp. now allowed for additional insights into clustering of secondary metabolite genes complemented by Cytochrome P450s. In the *Trichoderma* Cypome (the genome content of Cytochrome P450 encoding genes), 12 specific families of this functional group were detected (Chadha et al., 2018).

## Intriguing news about well-known model *Trichoderma* species

In recent years, improvement of the quality of genome sequencing was subject to intensive research. The genome of *T. reesei* QM6a was subjected to proximity ligation scaffolding (Jourdier et al., 2017) using the GRAAL software tool (Marie-Nelly et al., 2014), which already yielded an improvement compared to the initial assembly (Martinez et al., 2008). Approximately at the same time, long read sequencing using the PacBio or Nanopore (ONT; flowcell 10.4) technologies (supported by Illumina short read sequencing to enhance quality) was increasingly used to improve the quality of genome sequences and so called “gold-standard” genomes became available for many organisms. For *T. reesei* QM6a (Li et al., 2017), this high quality sequence revealed numerous sequencing errors, indels and inversions in older genome assemblies (Martinez et al., 2008; Marie-Nelly et al., 2014; Jourdier et al., 2017) and yielded more than 1000 previously undetected genes. Telomer-to-telomer sequences became available for all seven chromosomes along with the sequence of the mitochondrial genome which is identical with that published previously (Chambergo et al., 2002), confirming the high quality of the sequence. Additionally, a comparison to the RutC30 genome indicates that the number of translocation in this strain is lower than reported earlier (Seidl et al., 2008; Peterson and Nevalainen, 2012). The long reads obtained with third generation sequencing even allowed for correct sequencing of the highly AT-rich centromere region, in which several genes were detected (Li et al., 2017).

Since the achievement of sexual development under laboratory conditions, this process is subject to detailed investigation for important determinants as well as its consequences in the *Trichoderma* genomes (Schmoll, 2013; Hinterdobler et al., 2020b). In the course of investigation of recombination during meiosis, high quality genome sequences of CBS999.97(MAT1-1) and CBS999.97 (MAT1-2) (Lieckfeldt et al., 2000; Seidl et al., 2009) were prepared by third generation sequencing (Li et al., 2021a). Like QM6a, they also have seven chromosomes. As RIP (Gladyshev, 2017) is operative in *Trichoderma*, the CBS999.97 genomes only contain 62 transposable elements (Li et al., 2021a). Phenotypes of QM6a and CBS999.97 strains show considerable differences and their progeny are unexpectedly diverse (Seidl et al., 2009; Li et al., 2017). Accordingly, a very high number of SNPs and indels (ca. 1 Mio) were detected between QM6a and the CBS999.97 strain, but only around 2700 between CBS999.97(MAT1-1) and CBS999.97(MAT1-2). However, during analysis, problems with “difficult to align” sequences were observed and therefore the specifically developed software tool TSETA (TGS to Enable Tetrad Analysis; (Liu et al., 2021)) was used, which then identified around 6 fold more alterations between QM6a and CBS999.97 with sizes of up to 61 kb (Li et al., 2021a). Interestingly, for QM6a more than 3000 nucleotides were subject to RIP, while in CBS999.97 (MAT1-1) this was the case for only 92 nucleotides (Li et al., 2021a).

Although QM6a represents a nature isolate, it still carries an important mutation in its genome: sexual development was hampered by female fertility (Seidl et al., 2009). Ten rounds of backcrossing of QM6a with the fertile CBS999.97 allowed for narrowing down the genomic locus which contains the mutation (Schmoll et al., 2013). Subsequently it could be confirmed that the MAPKinase scaffold protein HAM5, which is encoded by a gene in this very locus, shows several mutations causing frame shifts and lead to an unfunctional gene, which renders QM6a female sterile (Linke et al., 2015; Tisch et al., 2017). This knowledge now enables a complementation of the defect to facilitate strain improvement by breeding. However, QM6a and recombinant strains derived from it can now also be used as a female sterile test strains to evaluate male and female fertility and knowledge-based crossings to remove the HAM5 defect serve to investigate mating in homozygous crosses (Hinterdobler et al., 2020b).

## Delving into the past – elucidation of the genetic basis of early and later random mutants

Since the advent of genome sequencing, the quest to elucidate the characteristics of high performance mutant strains of *Trichoderma* continues (Seidl and Seiboth, 2010). The number of mutations in these strains was greatly underestimated prior to knowledge on the genomes and hence, although many crucial functions of the mutated genes are already known (Seidl and Seiboth, 2010; Peterson and Nevalainen, 2012; Bischof et al., 2016), there are still numerous genes left, which are altered, but without deeper knowledge on their function or contribution to the phenotype of the respective mutant. Nevertheless, there was quite some progress in recent years.

One of the most important topics upon investigation of random mutants is gaining insight into the mechanism of cellulase regulation, both because of their relevance as homologously produced enzymes in *Trichoderma* and due to the efficiency of their promotors for heterologous protein production (Gudynaite-Savitch and White, 2016; Paloheimo et al., 2016; Arbige et al., 2019).

The random mutant strain QM9978 produces cellulases at a very low basic level, but cannot be induced under usual cellulase inducing conditions (Torigoi et al., 1996) and hence was used for comparative studies for decades (for example (Zeilinger et al., 2003; Schmoll et al., 2004)). Finally, in 2017, the defect of QM9978 was identified due to genome sequencing (Ivanova et al., 2017). Comparison with QM6a revealed 43 mutations, among them a translocation between chromosomes V and VII upstream of the transcription factor gene *vib1*, which caused the lack of cellulase induction in QM9978 (Ivanova et al., 2017). The homologue of VIB1 was previously reported as a link between glucose signaling and carbon catabolite repression and to be involved in regulation of plant cell wall degrading enzymes in *N. crassa* (Xiong et al., 2014). While overexpression of VIB1 did not increase cellulase gene expression in *T. reesei* (Ivanova et al., 2017), the same strategy led to significantly increased secreted cellulase activity in *T. orientalis* EU7-22 (Han et al., 2020).

The early random mutant *T. reesei* RUT C30 (Peterson and Nevalainen, 2012) is the most extensively studied mutant strain, as it serves as parental strain for many industrial production strains. Moreover, the considerable genomic rearrangements in this strain (Seidl et al., 2008) provided numerous hypothesis for strain improvement to be tested. Although quite some details on the basis for enhanced cellulase production of this strain are already known (Peterson and Nevalainen, 2012), it is still subject to research with the most recent finding, that the truncation of CRE1 present in RutC30 turns the repressor into an activator (Rassinger et al., 2018). Re-assembly and re-annotation of its genome showed diverse chromosomal rearrangements (Jourdier et al., 2017). The industrial application of derivatives of RUT C30 is also the reason for this strain to be used as parental strain for gene regulation studies. However, in this case, it has to be kept in mind that the numerous mutations of RUT C30, which also cause a considerably altered physiology including decreased growth and sporulation as well as weakened cell wall stability, do not allow for a reliable generalization of functional characteristics of individual genes to *Trichoderma* as a whole.

A less well known cellulase negative strain is *T. reesei* QM9136, which cannot grow on cellulose or form cellulases, but otherwise has a normal phenotype (Mandels et al., 1971; Nevalainen and Palva, 1978). In this strain, a frameshift mutation in the crucial cellulase transcription factor XYR1, which causes a truncation by 140 amino acids is responsible for the defect in cellulase production (Lichius et al., 2015). Additionally, 14 mutations have been detected which are likely irrelevant for cellulase production.

A descendant of the cellulase overproducer QM9414, which was subjected to several more mutagenic rounds is *T. reesei* PC-3-7 (Kawamori et al., 1985). Sequencing of this strain and comparison with the QM6a genome sequence yielded 260 SNPs in PC-3-7, of which most were located in non-coding regions. However, also in the important cellulase regulator genes *ace1* and *cre1* SNPs were detected. The SNP in CRE1 at amino acid 78 indeed caused a decrease in binding affinity of CRE1 to the *cbh1* promotor, which in turn negatively affected cellulase production (Porciuncula Jde et al., 2013).

## Bioprospecting in genomes of nature isolates

Investigation of the genome of novel biocontrol agents or biofertilizers is becoming increasingly interesting. On the one hand, these organisms are being distributed in nature in considerably larger amounts than would occur naturally. On the other hand, they can be sources for novel bioactive molecules (Karwehl and Stadler, 2016). In both cases the metabolic competences of a microbe can be a benefit or a threat to humanity. Especially the widespread application of biocontrol agents in the form of spores in low income countries raised questions as to the safety for farm workers there, but also the burden on consumers as well as nature in general (Konstantinovas et al., 2017; Hatvani et al., 2019). Clinically relevant *Trichoderma* strains are assumed to be limited to certain species (Kredics et al., 2003; Sandoval-Denis et al., 2014), but detailed knowledge on relevant secondary metabolite gene clusters is important to rule out selection of a potential harmful isolate for commercial application in agriculture. However, fungi including *Trichoderma* represent a potential source for valuable compounds to fight cancer or the counteract microbial resistance against common antibiotics (Viglas et al., 2021).

In all these cases detailed knowledge on the gene content of a given strain is crucial for knowledge based decisions on application (for an overview on recent genome level analyses of Trichoderma spp. aimed at biocontrol issues see **Table 1**). Additionally, a higher number of available genome sequences will enhance specificity of developed methods to detect biocontrol agents in agriculture (Kabani et al., 2002; Kredics et al., 2018), especially if identification of individual strains of a species is required – for example for following up distribution and habitat colonization of a biocontrol agent in the field. Unfortunately, genome level studies on health issues due to distribution of *Trichoderma* as biocontrol agents or of clinical isolates are still very rare.

**Table 1:** Recent genome level analyses of *Trichoderma* spp. targeted at biocontrol related issues.

Species	Strain	Topic	Key findings	Reference
*T. harzianum*	B97	biocontrol	Genes with non-synonymous SNPs compared to the reference strain are enriched in metabolic functions including secondary metabolism and DNA repair	(Compant et al., 2017)
*T. atrobrunneum*	ITEM 908	biocontrol	abundance of genes encoding CAZymes, secondary metabolite-, peptaibole- epidithiodioxopiperazine- and siderophore producing proteins is comparable to other *T. harzianum* complex associated species	(Fanelli et al., 2018)
*T. asperelloides*	T 203	mycoparasitism	genome announcement only	(Gortikov et al., 2022)
*T. virens*	FT-333	biocontrol, defense and nutrient utilization	gene content related to reactions to the environment varied compared to *T. virens* Gv29-8 and to other *Trichoderma* species	(Kuo et al., 2015)
*T. gracile*	HK011-1	biocontrol	antagonistic activity against several pathogens, gene annotation provided, secondary metabolite clusters detected	(Li and Liu, 2022)
*T. virens*	M7	biocontrol	deletion of 250 kb of the genome in five locations with 71 predicted genes	(Pachauri et al., 2020)
*T. koningiopsis*	UKM-M-UW RA5	biocontrol against *Erwinia mallotivora*	fungi controlling *E. mallotivora* identified, potential secondary metabolite pathways underpinning the antimicrobial properties of three antagonistic strains delineated	(Tamizi et al., 2022)
	UKM-M-UW RA6			
	UKM-M-UW RA3a			
*T. afroharzianum*	T11-W	control of nematodes and fungal plant pathogens	PacBio genome sequencing, annotation and basic comparative analysis to the high-quality genome of *T. reesei* QM6a	(Zhou et al., 2020)
*T. cyano-dichotomus*	TW21990-1			
*T. afroharzianum*	BFE349	mycoparasitism	genome announcement only	(Landeis and Schmidt-Heydt, 2021)
*T. asperellum*	TAIK1 TAIK4 TAIK5	biocontrol and plant growth promotion	genome announcement only	(Kannan et al., 2022)

Besides biocontrol and health related targets, also novel enzymes still remain an important commercial topic for which Trichoderma spp. are valuable (for an overview on recent genome level analyses of Trichoderma spp. aimed at bioprospecting see **Table 2**). Consequently, bioprospecting for novel, more efficient enzymes under diverse conditions – as required for various applications in industry – are still actively sought (enzymes reviews (Wilson, 2009; Singh et al., 2015; Arnau et al., 2020)).

**Table 2:** Recent genome level analyses of *Trichoderma* spp. targeted at bioprospecting of enzymes and bioactive compounds.

Species	Strain	Topic	Key findings	Reference
*T. harzianum*	B13-1	lipolytic activity	50 putative lipases detected; lipase gene inducable with olive oil identified	(Canseco-Perez et al., 2018)
*T. koningiopsis*	POS7	cellulase production in solid state fermentation	genome announcement only	(Castrillo et al., 2017)
*T. hamatum*	YYH13	cellulase production - variations in different strains	thirteen functionally important genes are under positive selection in the higher producing strain, 15 protease families are different and 10 further families of enzyme functionalities are subject to stronger positive selection	(Cheng et al., 2017)
*T. hamatum*	YYH16			
*T. simonsii*	GH-Sj1	asparaginase production	seven putative asparaginase-encoding genes detected, three of them are significantly upregulated under conditions enhancing asparaginase activity	(Chung et al., 2021)
*T. harzianum*	IOC-3844	biomass degradation	genes located in vicinity of those encoding biomass degrading enzymes were identified	(Crucello et al., 2015)
*T. harzianum*	T6776	enzymes for biofuel production	CAZymes identified, transcript levels analyzed (cellulose, lactose, sugar cane bagasse), phylogenetic analysis of AA9, CE5 and GH55 families showed high functional variation	(Ferreira Filho et al., 2017)
*T. harzianum*	IOC-3844	cellulose degradation	PacBio long read sequencing for genome, annotation, *clr2* is differentially expressed between glucose and cellulose, regulation network inferred	(Ferreira Filho et al., 2020)
*T. viride*	J1-030	sesquiterpene production	a novel sesquiterpene synthase was identified and characterized and the biosynthetic products of this enzyme were determined	(Sun et al., 2019)
*Trichoderma* sp. *(harzianum complex)*	MMS1255	peptaibol production	Pentadecaibin production associated with biosynthetic gene and antimicrobial activity detected	(van Bohemen et al., 2021)

Importantly, numerous additional newly sequenced genomes of the genus *Trichoderma* became available in JGI mycocosm (https://mycocosm.jgi.doe.gov/mycocosm/home) recently, which are not specifically described here.

## Mitochondrial genomes of *Trichoderma*

Mitochondria represent the powerhouse of every eukaryotic cell and are responsible for the production of ATP through the oxidative phosphorylation pathway and the aerobic citric acid (TCA) cycle and biosynthesis of metabolites like amino acids (Medina et al., 2020). Moreover, besides respiratory metabolism and energy production, mitochondria are also relevant for senescence during the cell cycle and maintenance of ion homeostasis (Basse, 2010; Chatre and Ricchetti, 2013). The mitogenome was shown to play a role in fungal virulence and emerged as an important target of fungicides (Medina et al., 2020).

In contrast to other cell organelles, mitochondria have their own genome, which can be present in multiple copies, also depending on growth conditions and is capable of independent replication and inheritance (Burger et al., 2003). Mitochondrial genomes have their own codon usage (Medina et al., 2020) and are highly variable, also with the extent of introns and their different sizes (Burger et al., 2003; Aguileta et al., 2014). Both high conservation of intronic sequences and their location within genes and species-specific introns were detected in Hypocreales, hence indicating an origin from a common ancestor as well as an alternative mechanism for intron evolution/transfer (Fonseca et al., 2020). Comparative analyses further showed a correlation between mitogenome length and the number and size of non-coding sequences in the mitochondrial genome of Hypocreales (Fonseca et al., 2020). Detailed sequence analysis revealed several cases of HGT of bacterial genera to fungi (Megarioti and Kouvelis, 2020). The different distribution of heavier and lighter nucleotides in the two strands of the mitochondrial genome enables their isolation by differential centrifugation (Garber and Yoder, 1983; Chambergo et al., 2002). The introns can contain so called homing endonucleases responsible for intron splicing (Megarioti and Kouvelis, 2020), of which the classes of LAGLIDADG and GIY-YIG are present also in fungal mtDNAs (Belfort et al., 2002; Stoddard, 2014). Such a gene conversion can happen through intron invasion and leads to altered size and composition of the mitochondrial genome (Wu and Hao, 2019; Medina et al., 2020). In fungi, mitochondrial genomes can be circular or linear with sizes from little more than 10 kb up to 200 kb (Pramateftaki et al., 2006; Losada et al., 2014; Liu et al., 2020), although the typical mitochondrial genome is much smaller. As for evolution of mitochondrial genomes, they were found to evolve more slowly than their nuclear genomes (Clark-Walker, 1991; Gaillardin et al., 2012), despite the high variability of non-coding regions.

In some cases, transfer of mitochondrial genes from the mitochondrial genome to the nuclear genome was observed and concerns genes like *nad5*, while for other genes no transfer events were detected (Fonseca et al., 2020; Medina et al., 2020). Over evolutionary time, a considerable number of the initially endosymbiotic genes were transferred to the nuclear genome and mitochondrial proteins are encoded by the nuclear genome and transported to the mitochondria (Burger et al., 2003). In the order Hypocreales, also duplication of mitochondrial genes is a common phenomenon as among the 17 core genes, only *atp8*, *atp9* and *cox3* were not detected in the respective nuclear genome (Fonseca et al., 2020).

The interest on research towards mitochondrial genomes remained very low with until recently just very few mitochondrial genomes published for the genus *Trichoderma* (Kwak, 2020; Kwak, 2021). In the last few years, with the advent of efficient and affordable genome sequencing methods, especially third generation sequencing, the numbers increased. Mitochondrial genomes in the Hypocreales are circular and range from about 24 kb to more than 100 kb in size (Fonseca et al., 2020). Thereby, the size of introns contributes to the variance of genome sizes (Medina et al., 2020).

**Figure 3:**
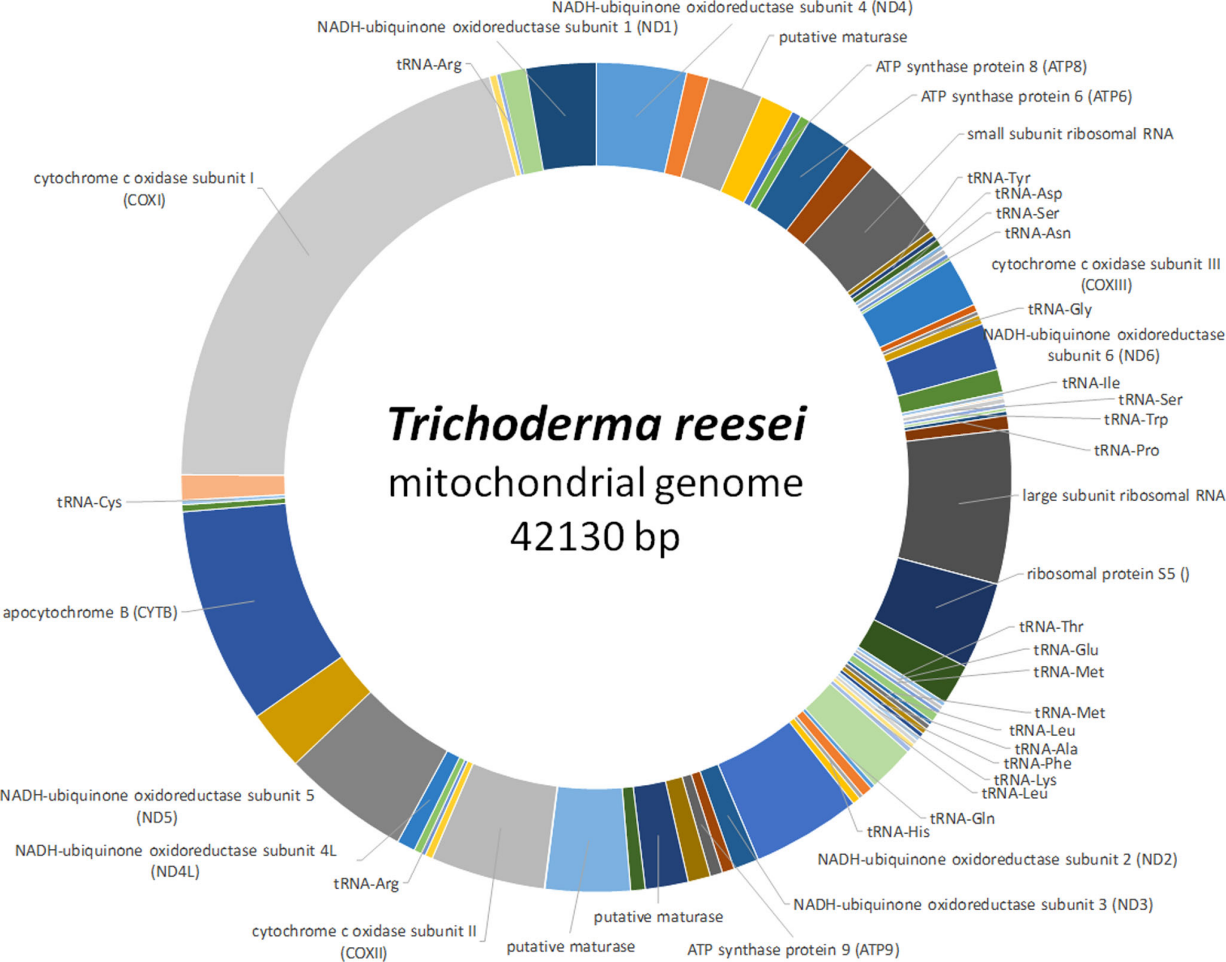
Schematic representation of the mitochondrial genome of *T. reesei*. The complete mitochondrial genome of *T. reesei* (GenBank accession number NC_003388) is shown along with encoded genes (Chambergo et al., 2002). Areas without label represent intergenic regions.

Already in the 1990s, the relevance of mitochondrial functions for physiology of *Trichoderma* was investigated, which revealed a significant impact on cellulase production, in that a low oxygen tension negatively influenced their biosynthesis (Abrahao-Neto et al., 1995). This sensitivity of *T. reesei* to the functional state of mitochondria was subsequently associated with the 5’ region of the major cellulase gene, *cbh1* (Carraro et al., 1998). A few years thereafter, the mitochondrial genome of *T. reesei* (**Figure 3**) was published (Chambergo et al., 2002). In *T. reesei* a role in oxidative stress resistance was proposed for mitochondria as well (Wang et al., 2015a). Recently, third generation sequencing yielded an update of mitochondrial genomes in *T. reesei*. The female sterile MAT1-2 strain QM6a (Li et al., 2017) was sequenced along with both mating types of *T. reesei* CBS999.97 (Seidl et al., 2009), which showed strikingly different sizes of the mitochondrial genomes yet a similar set of genes essential for mitochondrial functions (Li et al., 2021a). The mitochondrial genome of QM6a has 42 kb, while that of CBS999.97 (MAT1-1) has only 39 kb and only shares 75% of nucleotide sequence identity with that of QM6a. In contrast, the mitochondrial genomes of CBS999.97 (MAT1-1) and CBS999.97 (MAT1-2), which arose from the same crossing event (Seidl et al., 2009), are identical except for only six SNPs, hence reflecting maternal inheritance (Li et al., 2021a). Moreover, the mitochondrial genome sequences of *T. atroviride* P1, *T. asperellum* FT-101, *T. virens* Gv29-8 and *Tvirens* FT-333 vary considerably in size and analysis of high quality genomes of these strains indicates that mobile genetic elements played key roles in shaping the mitochondrial genomes in *Trichoderma* (Li et al., 2021b). Also three nuclear encoded mitochondrial sequences (NUMTs) were detected in *Trichoderma*, which are all located within an AT-rich block, which suggests that filamentous fungi and mammalian cells may have an evolutionarily conserved origin of NUMTs (Tsuji et al., 2012; Li et al., 2021b).

The mitochondrial genome of *T. harzianum* HB324 is circular and has a size of 32kb, with a GC content of 28% (Fonseca et al., 2020), which is in accordance with a generally high AT content in fungal mitogenomes (Medina et al., 2020). Fourteen genes associated with oxidative phosphorylation, 28 tRNA encoding genes as well as two ribosomal RNAs were detected in addition to a few hypothetical genes (Fonseca et al., 2020). As in many ascomycetes (Medina et al., 2020) all the genes were encoded on the same DNA strand in *T. harzianum* HB324. A comparison with other *Trichoderma* mitochondrial genomes showed a considerable variation in their structure and size even within the genus (Fonseca et al., 2020).

Another *T. harzianum* strain, CBS226.95, has a considerably smaller circular mitochondrial genome with only around 28 kb (Kwak, 2021). This mitogenome was further predicted to have evolved earlier than other *Trichoderma* species’ mitogenomes. It was further proposed that the evolution of *Trichoderma* mitochondria is influenced by their adaptive diversification depending on the diverse habitats from which *Trichoderma* strains were isolated concerning oxygen availability like soil, wood or living plants and fungi (Kwak, 2021).

*Trichoderma atroviride* ATCC26799 has a mitochondrial genome of 33 kb comprising the usual content of mitochondrial genes in the genus (Kwak, 2020). Gene order in the mitochondrial genomes of five *Trichoderma* spp. (*T. reesei* QM9414 (GenBank accession number AF447590), *T. asperellum* B05 (NC_037075), *T. hamatum* (MF287973), and *T. gamsii* KUC1747 (KU687109) is highly conserved, while intergenic regions, nucleotide composition bias, number of protein coding sequences and size of mitochondrial genomes was more variable (Kwak, 2020). Recently, also a mitochondrial genome of *T. simonsii* was reported (Chung et al., 2022), which clusters with several other *Trichoderma* mitogenomes including *T. cornu-damae* (Genbank accession number MW525445), *T. lixii* (NC_052832) and *T. hamatum* (NC_036144), most of which are not yet described in detail.

## Mycoviruses of *Trichoderma*

Viruses are able to infect living organisms from bacteria to humans and are present in fungi as well (Myers and James, 2022). Viruses of fungi have been known for more than 5 decades (Ghabrial et al., 2015), but the detection of them in *Trichoderma* spp. happened quite recently. In most cases, infection of a fungus with a virus does not change the phenotype (Ghabrial and Suzuki, 2009; Son et al., 2015), which is a likely reason that viruses in *Trichoderma* did not receive much attention so far. Nevertheless, there are some intriguing examples of mycoviruses considerably altering physiology and organismic interactions: If *Sclerotinia sclerotiorum* is infected with the small DNA mycovirus SsHADV-1, it turns from a pathogen of *Brassica* spp. into a beneficial endophyte due to downregulation of major pathogenicity factor genes (Zhang et al., 2020). Even more fascinating is the three-way symbiosis of the endophytic fungus *Curvularia protuberata*, which allows its host plant *Dichanthelium lanuginosum* to grow at high temperatures only if it is infected by the mycovirus CThTV (Marquez et al., 2007). Although it was initially thought that mycoviruses have a relatively narrow host range, detection of a virus first described in *Botrytis porri* in *B. squalosa* and *Sclerotinia sclerotiorum* suggests that this is not the case and that mycoviruses can be transmitted between species (Wu et al., 2012; Nerva and Chitarra, 2021). Intriguingly, also mycoviruses from endophytes were found to replicate in a plant, although this phenomenon was so far only shown *in vitro*, not in nature (Nerva et al., 2017).

The model fungus for studying the interaction of mycoviruses with their hosts is *Cryphonectria parasitica*, the chestnut blight pathogen, in which infection by a so called hypovirus decreases virulence (Dawe and Nuss, 2013; Eusebio-Cope et al., 2015). Similar effects were shown in other plant pathogens, hence making mycoviruses an interesting subject to research towards biocontrol applications (Milgroom and Cortesi, 2004). In this respect, treatment of chestnut blight with mycoviruses exemplified the problem with such an application. No natural vectors are known for mycoviruses (Ghabrial and Suzuki, 2009; Son et al., 2015). They spread predominantly vertically by propagation *via* conidiospores, less efficiently *via* ascospores in ascomycetes (Ghabrial et al., 2015). Alternatively, infection occurs by hyphal anastomosis, which is limited by vegetative incompatibility, leading to programmed cell death if two incompatible fungi fuse (Daskalov et al., 2017). In case of multiple incompatibility groups in fungal population, spread of the viruses is very inefficient as is biocontrol in such a case (Xie and Jiang, 2014). The limited success of treatment of *C. parasitica* in the US compared to the imported strains in Europe is attributed to this problem (Myers and James, 2022). In *Trichoderma*, vegetative incompatibility was shown previously (Gomez et al., 1997), but is not yet sufficiently investigated to draw any conclusions as to the impact on biocontrol enhancements by mycovirus applications.

Another natural defense mechanism that limits infection of pathogens by mycoviruses is RNAi, meant to destroy intruding foreign nucleic acids (Segers et al., 2007). Interestingly, although mycoviruses often only have two genes encoded, they can counteract programmed cell death and vegetative incompatibility as well as RNAi (Hammond et al., 2008; Daskalov et al., 2017), which tips the balance again towards their benefit. Often, the presence of a mycovirus in a fungus causes lower growth rates which is interpreted as a lack of fitness (Ghabrial and Suzuki, 2009). Nevertheless, also co-evolution of mycoviruses with their hosts has been observed, although this observation cannot be generalized (Nerva and Chitarra, 2021).

**Figure 4:**
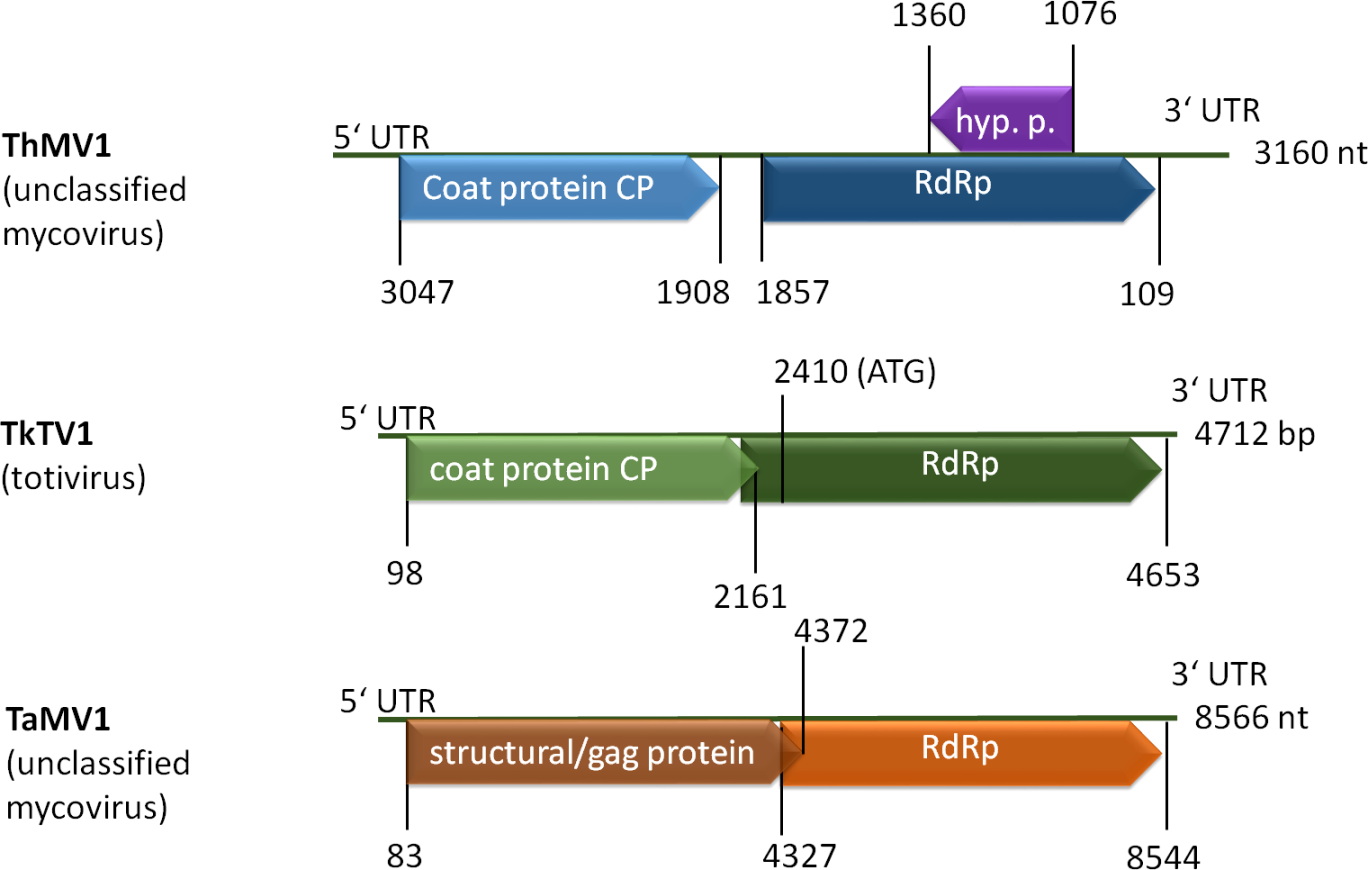
Schematic representation of selected mycovirus genomes. ThMV1 (Liu et al., 2019), TkTV1 (Khalifa and MacDiarmid, 2019) and TaMV1 (Lee et al., 2017) genomes comprise two ORFs coding for a coat protein (CP or GAG) and an RNA dependent RNA polymerase (RdRp). ThMV1 additionally comprises a small ORF encoding a hypothetical protein of unknown function.

In *Trichoderma*, the first mycovirus was described in 2009 (Jom-in and Akarapisan, 2009) and only a few followed thereafter. However, a study of more than 300 fungal isolates causing green mold disease revealed evidence for potential dsRNA mycoviruses of diverse groups in 32 isolates, indicating that mycoviruses are not uncommon in *Trichoderma* (Yun et al., 2016). The genomes of these mycoviruses are relatively small and they mostly encode only two proteins, a coat protein and an RNA dependent RNA polymerase (RdRp) (**Figure 4**). As reported from other fungi, mycoviruses often do not influence the phenotypes of their hosts or just have a minor impact on pyhsiology, which was also observed for several isolates from *Trichoderma* (**Table 3**).

**Table 3:** Reports on detection of mycoviruses in diverse *Trichoderma* species.

Species	Strain	Virus	Type	Key findings	Reference
*T. atroviride*	NFCF028	TaMV1	dsRNA	8 kb mycovirus with two open reading frames detected, member of a distinct species, unclassified	(Lee et al., 2017)
*T. harzianum*	137	ThBMV1	dsRNA	ThBMV1 clusters with other unclassified dsRNA mycoviruses	(Liu et al., 2019)
*T. asperellum*	JLM45-3	TaRV1	dsRNA	Virus, approximately 10 kb in size, with two open reading frames associated to a taxonomically unassigned mycovirus group, new family proposed as *Fusagraviridae*	(Zhang et al., 2018)
*T. atroviride*	NFCF394	TaPV1	dsRNA	TaPV1 belongs to the genus *Alphapartitivirus* in the family *Partitiviridae*. Virus cured strains did not show phenotypic alterations.	(Chun et al., 2018a)
*T. koningiopsis*	Mg10	TkTV1	dsRNA	TkTV1 represents a novel Totivirus of approximately 5 kb and is highly similar to a mycovirus from *Clonostachys rosea* isolated from the same sample. TkTV1 could infect both strains.	(Khalifa and MacDiarmid, 2019)
*T. harzianum*	M6	ThHV2	(+)ssRNA	The 14 kb mycovirus contains one large open reading frame with five conserved motifs and likely belongs to the proposed genus Alphahypovirus	(Chun et al., 2022)

In several cases however, presence of the mycovirus in the *Trichoderma* strain did cause phenotypic alterations, mostly enhanced mycoparasitic abilities:

A mycovirus in *T. harzianum* (ThMV1) was investigated in more detail and caused a decrease in biomass production, a slightly positive effect on plant health if *T. harzianum* was applied alone and somewhat better biocontrol activity against *Fusarium oxysporum* f. sp. *cucumerinum* in cucumber (Liu et al., 2019). The *T. atroviride* mycovirus TaMV1, a member of the proposed family *Fusagraviridae* does not impact conidiation or growth, but supported enhanced antifungal activity against the plant pathogen *Rhizoctonia solani* (Chun et al., 2020). For the *T. harzianum* mycovirus ThHV1, which shares similarity with betahypoviruses, an influence on mycoparasitism was shown in case of the presence of a defective genome. This mycovirus could be transmitted vertically to conidia, but could also infect another *T. harzianum* isolate as well as *T. koningiopsis* (You et al., 2019). The *T. harzianum* partitivirus 1 (ThPV1) enhances growth inhibition of co-cultured plant pathogens as well as glucanase activity (Chun et al., 2018b).

The still few reports on mycoviruses in *Trichoderma* indicate that investigation of mycoviruses and their interaction with the host as well as their influence on biocontrol of plant pathogens are emerging as an important new topic of research. Since it was shown that mycoviruses can have a broad host spectrum, transmission of a mycovirus from a pathogen to a mycoparasite like *Trichoderma* or vice versa is likely to be possible, yet the consequences for plant health, population structure and ecology are currently completely unknown. Additionally, the effects of mycovirus infections in *Trichoderma* are hardly predictable and loss of a mycovirus can easily happen during industrial cultivation, potentially leading to altered characteristics of the fungus. Consequently, it will be crucial to be aware of a potential mycovirus in a fungus if it is part of a commercial product in order to guarantee a stable, efficient product and knowledge on susceptibility of the fungus for mycovisuses abundant in the target pathogen will be beneficial.

In this respect, the increasing performance of next generation sequencing of transcriptomes (revealing RNA viruses) comes in handy, as a previous screening of available GenBank data shows (Gilbert et al., 2019). Detection of mycoviruses as a by-product of gene regulation studies is a worthwhile effort to gain important information on model organisms as well as production strains and biocontrol agents.

## Genomic aspects of mycoremediation with *Trichoderma*

As the awareness of the community towards a healthy environment increases, contaminated sites, which could previously hardly be treated efficiently, comes into focus (Febbraio, 2017; Ford et al., 2022a; Sharma et al., 2022). The genomes of *Trichoderma* spp., originating from diverse habitats represent a treasure trove for the quest for enzymes and metabolic competences to detoxify or even mineralize dangerous chemicals (Tripathi et al., 2013; Zafra and Cortes-Espinosa, 2015; Repas et al., 2017; Mishra et al., 2021) and *Trichoderma* species are well known for their efficiency in remediation of soil and water pollution (Harman et al., 2004b). Fungi are generally very potent organisms for bioremediation (Kour et al., 2021), even outperforming bacteria (Dell'Anno et al., 2022). The decades-long application of *Trichoderma* spp. in biocontrol and in industry led to an in depth knowledge of their environmental safety (Nevalainen et al., 1994; Frisvad et al., 2018; Shenouda and Cox, 2021), which makes fungi of this genus prime candidates for application at natural contaminated sites with limited negative effects on the surrounding flora and mostly without the need for genetic modification. The chemical composition of pollutants is diverse, representing a considerable challenge for strain selection. In case of plastic degradation however, suitable enzyme classes for degradation of the respective structures can be defined beforehand (Verschoor et al., 2022).

Genome mining and omics analyses allow for delineating regulation pathways of suitable target enzymes, which is important for performance of a given strain, as not only the presence of degradative enzymes, but also their appropriate regulation under commercially viable conditions is crucial for applicability. This strategy is not yet routinely applied in *Trichoderma*, but in recent years, several interesting examples of detailed investigation of genomes, enzymes targeting certain pollutants and delineation of degradation pathways were reported.

*Trichoderma lixii* MUT3171 was isolated from a highly polluted environment and comparison of its genome was used to gain insight into potential degradation pathways of polycyclic aromatic hydrocarbons (PAHs) (Venice et al., 2020). Orthologues of genes encoding oxidoreductases, CAZymes and proteins responsible for biosurfactant biosynthesis were screened in 14 *Trichoderma* species. Additionally, also unique genes of MUT3171 including a quinoprotein alcohol dehydrogenase and a specialized mechanism of DNA repair were determined which may contribute to the ability of *T. lixii* to survive in this habitat (Venice et al., 2020). The dichlorodiphenyltrichloroethane (DDT) resistant *T. hamatum* strain FBL587 was investigated for its transcriptomic reaction to the pollutant (Davolos et al., 2021). This strain could degrade DDT and enhance degradation by *Cucurbita* phytoremediation. Especially Cytochrome P450 enzymes encoding genes were upregulated in the presence of DDT, but also transforming enzymes such as epoxide hydrolases, FAD-dependent monooxygenases, glycosyl and glutathione transferases as well as transporters (Davolos et al., 2021). The example of this detailed analysis shows how crucial genes for xenobiotic remediation can be narrowed down, which can represent an important step for strain screening and selection for future bioremediation purposes by fungal cultures or their isolated enzymes.

Currently, approaches to realize circular economy by valorization are in development – for example with *Trichoderma* spp. degrading waste biomass for production of metabolites (Shenouda and Cox, 2021; Shenouda et al., 2022), which should be extended to degradation of more problematic waste materials (Verschoor et al., 2022) like plastics or composites. In this respect the ability of adaptation of fungal strains to the substrate by crossing (Ashton and Dyer, 2016) can be a valuable tool for strain improvement towards plastics degradation and detoxification of pollutants. So far, only for *T. reesei* crossing was achieved under laboratory conditions (Seidl et al., 2009) and the method has successfully been applied to increase cellulase production by at least 10fold compared to RutC30 (M. Schmoll and S. Basyouni-Khamis, unpublished results). The availability of European nature isolates of *T. reesei*, which are sexually fertile (Hinterdobler et al., 2021a) is an important prerequisite to test a crossing approach for using plastics and/or their degradation products as carbon source to produce higher value chemicals and enzymes. Such a scheme of circular economy could serve as a blueprint for avoiding pollution and creating high value products with minimal pollution in the future, which makes further genome mining and efforts to achieve crossing with other *Trichoderma* spp. as well a worthwhile effort.

## Tools for genome screening and manipulation

Fungal genomes are constantly subject to manipulation – mostly of course in nature in order to achieve optimal adaptation to the habitat or just to enable survival if environmental conditions deteriorate. Sexual development represents the major strategy of nature to modify the genome, but also a valuable tool for strain improvement in the lab (see above). However, also artificial methods for genome manipulation of fungi were developed further in recent years.

Functional analysis of genes and whole pathways are especially important to understand the physiology of *Trichoderma* and consequently, strategies for increasing the ease and efficiency of genome manipulation are constantly optimized (Guangtao et al., 2009; Schuster et al., 2012; Chum et al., 2017). A more recent addition is the TrichoGate cloning strategy which is mainly an adaptation of the Golden Gate cloning system to *Trichoderma* (Nogueira-Lopez et al., 2019). Using this system, vectors for different promotors, deletions, protein localization studies and overexpressions are introduced along with a vector for *Agrobacterium* mediated transformation.

Also the SES (synthetic expression system), which was previously established for *Saccharomyces cerevisiae* (Rantasalo et al., 2016; Rantasalo et al., 2019) represents an interesting addition to the toolset for genome manipulation in *Trichoderma*, which is focused on protein expression (Rantasalo et al., 2018). In this system induction of protein synthesis involves two cassettes for low and constitutive production of a synthetic transcription factor, which activates a promotor in a second cassette with strong and tunable expression (Rantasalo et al., 2018).

### CRISPR – adaptation to Trichoderma and optimization

Traditionally, genome manipulation in *Trichoderma* spp. was done using protoplast transformation, electroporation, agrobacterium mediated transformation or biolistic transformation (Rodriguez-Iglesias and Schmoll, 2015; Schmoll and Zeilinger, 2021). However, in recent years, the versatile method of clustered regularly interspaced short palindromic repeat (CRISPR)-associated Cas9 (Hoof et al., 2018; Wang and Coleman, 2019; Rozhkova and Kislitsin, 2021) was also established for *Trichoderma*, particularly *T. reesei* (Liu et al., 2015; Zou and Zhou, 2021) and *T. harzianum* (Vieira et al., 2021) and subsequently optimized. Usually, the specific gRNA and the Cas9 protein are introduced as DNA into the host organisms which leads to constitutive exposure of the fungus to active Cas9. This can cause decreased viability and genome integrity of the host, but most importantly also unintended genome modifications. It was shown that *in vitro* assembly of Cas9 and gRNA prior to transformation of the nucleoprotein complex with a marker plasmid or the donor DNA was less prone to off target gene disruption than intracellular expression of Cas9 (Hao and Su, 2019; Rantasalo et al., 2019). Later on, the 5S rRNA promotor of *A. niger* was suggested for expression of the guide RNA, which enabled gene deletion using a donor DNA carrying only a 40 bp homology sequence and no selectable marker gene (Wang et al., 2021). Similarly, the promotors of two RNA polymerase III U6 snRNA genes were confirmed to be suitable for gRNA expression in *T. reesei* (Wu et al., 2020). Although the method of genome editing has become quite popular for modifications in fungi, the method *via* Cas9-CRISPR gRNA ribonucleoprotein complexes assembled *in vitro* is relatively low. Addition of chemicals like Triton X-100, inositol or benomyl led to increased efficiency in transformation as well as homologous integration (Zou et al., 2021). Nevertheless, the method is still relatively young and more improvements – for example as developed for *Saccharomyces cerevisiae* (Antony et al., 2022) – await adaptation and establishment in *Trichoderma*. Moreover, application CRISPR has not been established in several other *Trichoderma* species, which may be due to the capability of *Trichoderma* to clear their genomes of foreign DNA elements and hence further efforts are required.

### Tools for genome mining

Secondary metabolites of fungi are generally an important resource for novel pharmaceuticals and antibiotics, but can also represent a threat to human health (Karwehl and Stadler, 2016; Bhattarai et al., 2021; Juraschek et al., 2022). Therefore it is very important to tackle new ways of genome mining and investigation, as was done for Ribosomally synthesized and posttranslationally modified peptides, RiPPs, in *Trichoderma* (Vignolle et al., 2020). These compounds add a further way of biosynthesis to polyketides and non-ribosomal peptides in that they are encoded within a precursor and subsequently processed after posttranslational modification. However, evaluation of the biological function of RiPPs is still at its beginnings and roles in defense, deterring mycophagy, support of nutrient acquisition, competition, but also in symbioses (Ford et al., 2022b). Despite the relevance of for example alpha-amanitin or phallacidin, which are formed by *Amanita* spp., current screening software is focused on bacterial sequences and reports on biosynthesis in fungi is scarce (Kessler and Chooi, 2022). The multistep approach now presented (Vignolle et al., 2020) and including manual inspection yielded a wide range of RiPP candidates for *Trichoderma* spp., from 6 in *T. reesei* to 222 in *T. harzianum*, which indicates a considerable relevance of these compounds for physiology of *Trichoderma*. Together with the potential importance of novel RiPPs as bioactive substances, their biological relevance to fungi and especially *Trichoderma* warrants further investigation.

A novel tool for screening for essential biosynthetic genes was developed to enable determination of biosynthetically relevant genes in clusters versus those which are not needed for secondary metabolite production within the cluster – the so-called gap genes (Vignolle et al., 2021). The tool “FunOrder” (Vignolle et al., 2021) was also tested for *Trichoderma* and applies computational molecular co-evolution to distinguish between biosynthetic genes and gap genes. Thereby FunOrder facilitates efficient heterologous expression of biosynthetic gene clusters as well as functional analysis of the underlying biochemical pathways.

Generally, for screening for secondary metabolite clusters responsible for novel bioactive compounds in *Trichoderma* several software packages are useful (Rush et al., 2021). Besides the well-known tool antiSMASH (Blin et al., 2019), which detects biosynthetic clusters for secondary metabolites, the tool amPEPpy (Lawrence et al., 2021) can be used for screening for antimicrobial peptides.

## Author contributions

MoS drafted the manuscript, MiS edited the manuscript and all authors agreed on the final version of the manuscript.

## Funding

Work of MoS and MiS was supported by the Austrian Research Fund (FWF), grant P31464 to MoS.

## Conflict of interest

The authors declare that the research was conducted in the absence of any commercial or financial relationships that could be construed as a potential conflict of interest.

## Publisher’s note

All claims expressed in this article are solely those of the authors and do not necessarily represent those of their affiliated organizations, or those of the publisher, the editors and the reviewers. Any product that may be evaluated in this article, or claim that may be made by its manufacturer, is not guaranteed or endorsed by the publisher.

## Footnotes

1
https://www.marketsandmarkets.com/Market-Reports/biofungicide-market-8734417.html

2
https://www.mordorintelligence.com/industry-reports/global-fungi-based-agricultural-microbial-market
